# Diving Deep into the Data: A Review of Deep Learning Approaches and Potential Applications in Foodomics

**DOI:** 10.3390/foods10081803

**Published:** 2021-08-04

**Authors:** Lisa-Carina Class, Gesine Kuhnen, Sascha Rohn, Jürgen Kuballa

**Affiliations:** 1 GALAB Laboratories GmbH, Am Schleusengraben 7, 21029 Hamburg, Germany; lisa-carina.class@galab.de (L.-C.C.); gesine.kuhnen@galab.de (G.K.); 2 Hamburg School of Food Science, Institute of Food Chemistry, University of Hamburg, Grindelallee 117, 20146 Hamburg, Germany; 3 Department of Food Chemistry and Analysis, Institute of Food Technology and Food Chemistry, Technische Universität Berlin, Gustav-Meyer-Allee 25, 13355 Berlin, Germany; rohn@tu-berlin.de

**Keywords:** deep learning, machine learning, metabolomics, food authenticity, food fraud, shelf-life, peptide sequencing, mass spectrometry

## Abstract

Deep learning is a trending field in bioinformatics; so far, mostly known for image processing and speech recognition, but it also shows promising possibilities for data processing in food analysis, especially, foodomics. Thus, more and more deep learning approaches are used. This review presents an introduction into deep learning in the context of metabolomics and proteomics, focusing on the prediction of shelf-life, food authenticity, and food quality. Apart from the direct food-related applications, this review summarizes deep learning for peptide sequencing and its context to food analysis. The review’s focus further lays on MS (mass spectrometry)-based approaches. As a result of the constant development and improvement of analytical devices, as well as more complex holistic research questions, especially with the diverse and complex matrix food, there is a need for more effective methods for data processing. Deep learning might offer meeting this need and gives prospect to deal with the vast amount and complexity of data.

## 1. Introduction

**Figure 1:**
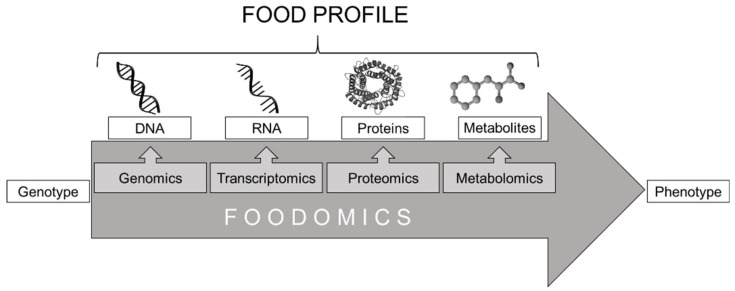
Scheme of the omics-cascade.

New challenges and the development or improvement of analytical methods in the last years come with the need for new approaches enabling a holistic way to evaluate food products [1,2]. Foodomics is not really a well-defined term being used to unite the analytical technologies and disciplines of the omics-cascade with research questions in food and nutrition [1,3]. Genomics, proteomics, and metabolomics are part of the so-called omics-cascade, comprising disciplines, technologies, and methodologies that are commonly used to describe the whole profile of food compounds [4]. Figure 1 illustrates schematically this cascade. These applications are often also called high-throughput technologies, producing a substantial amount of data [5,6,7]. Every discipline provides different information about the composition of the target. As the analytical date collected is steadily increasing, a new approach is the integration of a mathematical/bioinformatic point of view into foodomics. The first discipline of the omics-cascade is genomics and focuses on the investigation of the entire genome, meaning all genetic material of an organism based on the four bases DNA code with its 64 codons, allowing a countless number of sequences. Because of the low adaption to exogenous influence factors, the genome is predominantly stable and offers a valuable tool for the differentiation and identification of species.

Transcriptomics is the next discipline in the course of the omics-cascade and describes the analysis of the transcriptome, which includes mRNA, non-coding RNA, as well as small RNA in an organism [8]. The transcriptome is, unlike the genome, not stable and therefore, almost unsuitable for analytical applications regarding food investigations, because of the difficult dynamics [4]. The trancriptome leads to proteins. Proteomics describes the study of all proteoformes in a defined biological system [9]. Nowadays, it seems to be almost clear that the estimated number of genes (~100,000) is much higher than the anticipated number identified: (~20,300) [10]. Moreover, this means that at the protein level, the variations is much higher because of the encoded proteins, but also all kinds of follow-up modifications [11]. Similar to other biological matrices, investigating the proteome of a food product can be done with the following two strategies: On the one hand, the so called top-down approach focuses on the characterization of intact proteins [12,13,14]. In contrast, the bottom-up approach usually focuses on the peptides resulting from a proteolytic digestion of the proteins [13,14]. Both targets—proteins and peptides—are analyzed afterwards with mass spectrometry [14]. Metabolomics is the next discipline in the omics-cascade and focus on the identification and quantification of the whole metabolome [4]. This includes substrates and products of metabolic pathways and is directly associated to the so called phenotype [14,15]. So, it is obvious that the metabolome is heavily influenced by all kinds of exogenous factors. However, the metabolome enables an even more pronounced fingerprint of a system, as many compounds can be taken into account for an evaluation and a certain status of product at a certain timepoint can be estimated [4,5,6,7,14,16].

In the following, this review focuses primarily on proteomics and metabolomics, because those approaches are mainly mass spectrometry (MS)-based and the most prominent disciplines for differentiating food products with regard to identification (‘authentication’), but also characterizing the influence factors altering the phenotype (‘status’). The MS-based methodologies offer a basis for many chemometric applications, especially with regard to food analysis.

MS is the primary analytical technique to perform proteomics and metabolomics. It is a technique for additionally separating molecules (besides chromatography), but with regard to the mass-to-charge ratio (*m/z*) of an ion. However, in nearly all cases, MS-based investigations of food are coupled with liquid chromatography (LC) or gas chromatography (GC), where the analytes are additionally separated, with a certain clean-up, before entering the mass spectrometer [17]. There are two combination methods for those applications: on the one hand low-resolution mass spectrometry (LRMS) often provided as a triple-quadrupole as an analyzer and on the other hand high-resolution mass spectrometry (HRMS) with time-of-flight or similar detectors [12,18,19].

To describe the composition of food, HRMS technologies are applied preferably, as these instruments provide the most efficient results, facing different challenges related to the structure and quantity of molecules in natural products as well as processed food products [17,18].

HRMS makes it possible to also separate isobaric molecules [18]. Another impressive aspect of this technology is that HRMS enables screening and quantifying without a reference standard. In this context, a reference standard is the exact substance, which is the target of an investigation. LRMS needs those references to identify the analytes. With HRMS suspect screening and non-targeted applications can be performed, while not needing references, because of a precise mass-to-charge-ratio and the generating of other values like the collisions cross section (CCS) [18,20]. These reasons often led to the conclusion that the superior choice to perform non-targeted approaches of complex matrices is HRMS [12,18,19,21,22]. However, these different omics provide lots of data. Consequently, chemometric tools need to be developed and applied. The goal is to deal effectively with the large amount of data, to achieve different assignments like the prediction of shelf-life, identifying food fraud, proving food authenticity, and evaluating food quality, in general [23,24]. The novel deep learning approaches might provide an adequate tool for answering holistic food-related questions in the future and also grant even more possibilities than the traditional applications.

## 2. Chemometrics, Artificial Intelligence, and Machine Learning

When dealing with this emerging topic, primarily some terms and concepts need to be introduced to enable strategies and protocols for chemometrics. When starting with the term Chemometrics itself, this has been defined by the International Union of Pure and Applied Chemistry (IUPAC) as “The science of relating measurements made on a chemical system or process to the state of the system via application of mathematical or statistical methods.” [25,26]. This definition includes the combination of the disciplines chemistry, mathematics, and computer science with the focus on the generated data.

**Figure 2:**
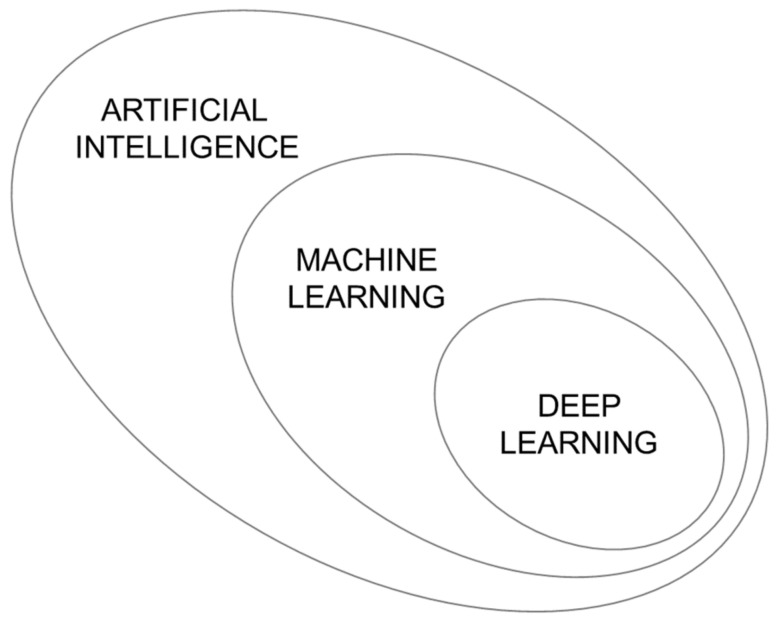
Relationship of artificial intelligence, machine learning, and deep learning [30].

Artificial Intelligence (AI) is an expression many stumble across regularly, but it remains unclear what it really means or comprises. IUPAC defined AI as “The capability of a machine to perform human-like intelligence functions, such as learning, adapting, reasoning and self-correction.” [27]. Machine Learning (ML) is considered as a subclass of AI, covering the methods of detecting and learning. Patterns need to be determined and learning leads to optimization, and using these enables decision-making or predicting future outcomes [28]. Deep Learning (DL) in turn is a certain form of ML, which is presented in more detail in the next section. The relationship between AI, ML, and DL is shown in Figure 2. A further term that is often mentioned in relation to the others is Data Mining (DM). DM is a step of knowledge discovery in databases (KDD). The tools used for DM are often ML tools. DM or KDD general focus on the knowledge discovery including storage, access of data, as well as visualization; all in all, providing a workflow for an evaluation process [29].

ML methods are categorized into supervised, unsupervised, and reinforcement learning. Supervised learning is based on labeling the training sets with the desired output. It is the most common used technique. Common tasks in supervised learning are regression and classification tasks. While classification tasks provide a categorical output, regression provides real-valued outputs. Unsupervised learning can be used for the discovery of patterns in datasets (‘fingerprints’), where no labelling of data is given in advance. Reinforcement learning is based on reward or punishment signals [28].

There are some issues that should be considered when ML methods are applied. The training of supervised models is based on labelled data. These are processed and fitted to improve performance. Accordingly, the efficiency of the model depends on the used dataset. Limitations are inadequate small datasets, non-representative data, and an insufficient quality of the datasets. While the model is trained, overfitting is a problem as well. Overfitting means that the model performs well with the training data but difficulties are occurring in generalizing or adapting the patterns observed to new data. Overfitting can be caused by different reasons. One might be that the model has been trained using the same data with too many repetitions. Another reason for overfitting is models which are too complex for the given task. Moreover, it can also be caused by datasets that are insufficient in representing the generality. Underfitting on the other hand results from models that are too simple for a rather complex topic [31,32].

Some commonly used chemometric and ML models in foodomics are principle component analysis (PCA) [33], partial least square-discriminant analysis (PLS-DA) [34], support vector machine(s) (SVM) [30], random forest (RF) [35], decision tree (DT) [36], and k-nearest neighbors (kNN) [37,38].

## 3. Deep Learning

**Figure 3:**
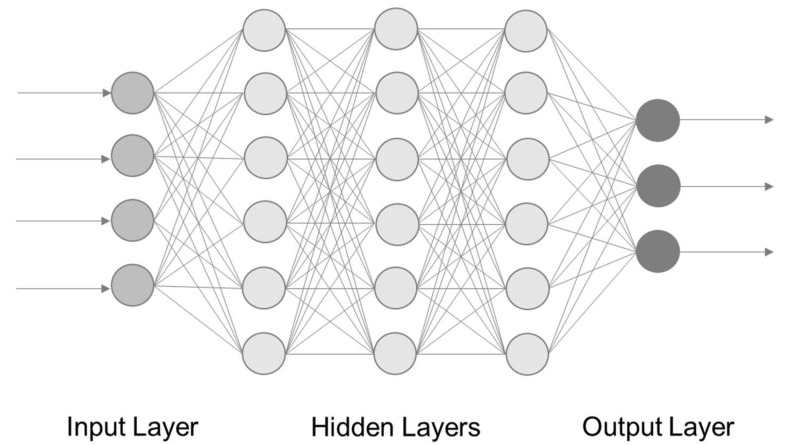
Architecture of a feedforward neural network with four input neurons, and three output neurons, and (here exemplarily three) hidden layers [40].

Conventional machine-learning-systems need manual feature extraction, whereas DL systems learn these features from the trainings data [39]. DL is learning of representation of data through layers of neurons [30]. These neurons are structured in form of Neural Networks (NN). As the name indicates, NN consist of artificial neurons. The first NN are based on computational concepts that are structurally similar to biological neurons and on how they might work together as a network [31]. Artificial neurons receive one or more inputs, which can activate a neuron to give an output. Figure 3 shows the structure of a simple NN, also called multilayer perceptron (MLP) [30]. 

It is a feedforward neural network (FNN) that consists of three main components: the neurons (also called nodes), the connection between these neurons, and the layers. The layers and thereby the neurons are divided into input, hidden, and output [39]. The input neurons receive the raw data. Each of the varying number of hidden layer of neurons takes the sum of the outputs of the preceding layer as an input. This input goes through an activation function, generating output according to the value of the input [39]. DL is not equal to NN, although it is often used in a similar way.

The differentiation between deep and shallow NN are based on the number of hidden layers, but with no defined number of them [31]. NN are categorized in feedforward NN (FNN) and recurrent NN (RNN). FNN are acyclic, as the data stream goes straight through each layer from input to output [41]. In the previous section and Figure 3, a simple type of FNN was described; a special kind of FNN that should be pointed out is the convolutional neural network (ConvNet, CNN). It consists of one or more convolutional layers followed by a pooling layer. While the convolutional layer detects features in the input matrix, the pooling layer reduces the dimension. After convolutional and pooling layers are applied, usually fully connected layers are used as well [39]. Recurrent neural networks (RNN) on the other hand, are distinguished by feedback connections. Due to the feedback connection, the output of the neuron is not only influenced by the current input of the neurons in the preceding layer, but also by inputs from previous timepoints. The so-called hidden state of the neurons provides a kind of memory from the previous layers. Therefore, RNN are strong for processing sequential data [31,42]. The long short-term memory (LSTM) is a variant of RNN, coping with the challenge of the loss of long-term information in conventional RNN. The LSTM features so-called memory cells with three gates: input, output, and forget gate. These enable the cells to extract and save important input in long-term aspects [43].

The most used learning technique in DL is supervised [39]. Supervised, unsupervised, and reinforcement learning have already been described above. Supervised learning uses backpropagation. As already mentioned, labelled datasets are needed. The data is split into training and testing data. The bigger part is used for the training of the model. Initially, the output of a small dataset is calculated, then the error in comparison with the desired output is considered, and subsequently the weights and biases are adjusted. This is performed multiple times until the functions reaches a local minimum [39,44].

For the application and programming of DL algorithms, the most popular programming languages are Python [45], R [46], and MATLAB [47].Frameworks like TensorFlow [48] and PyTorch [49] can be used, making the application more accessible, due to the simplified integration of models and various available tutorials, even with a limited background knowledge in informatics. Last but not the least, the improvement of graphic processing units contributed to the success of DL in recent years. These enabled the acceleration of the NN training [39,41].

## 4. Food Fraud and Food Authenticity

**Figure 4:**
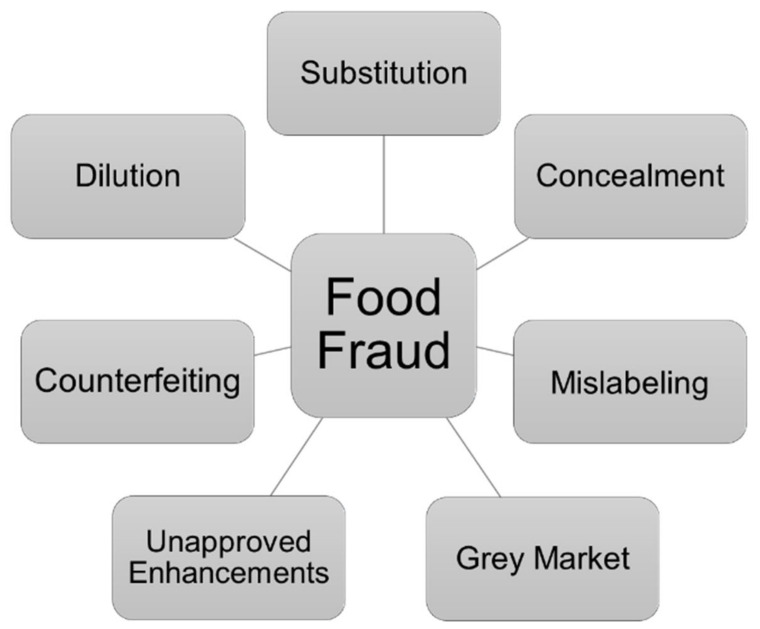
Subtopics of food fraud [61,62,63].

Food Fraud, Food Crime, Food Adulteration, and Food Terrorism are just a few terms to describe different food safety issues that have been especially associated with authentic food, which refers to a certain (production) technology, origin, or other specificities. The European Commission describes food fraud as “any suspected intentional action by business or individuals for the purpose of deceiving purchasers and gaining undue advantages therefrom, in violation of rules referred to in Article 1 (2) of Regulation (EU) 2017/625 (the agri-food chain legislation)”. Food fraud includes, referring to the Food and Drug Administration, economically motivated adulteration or the concept of food counterfeiting [50,51]. This means that once the intentional violation against the food law is committed, in most cases for achieving an economic or financial benefit, it is a matter of food fraud, and the consumers are at risk of being cheated or even consumer’s health is in danger [50,52]. There are different types to perpetrate food fraud as shown in Figure 4. One subtopic is the non-approved enhancement of some food products. An example for that is the so called “melamine scandal 2008” in China, where melamine was added to infant formula and other food materials to enhance the protein value by mimicking proteins with melamine as intense nitrogen-containing compound [53,54,55,56]. Another subtopic of food fraud is “substitution” (Figure 4). Here, examples are the substitution of mineral oils in specific vegetable oils or the mixture of high-priced vegetable oils and cheaper vegetable oils to gain financial profit [38,57].

Many more examples of food fraud incidents in the last couple of years could be named, like dioxin in eggs and chicken meat or the “horse meat scandal” in Europe 2013 [55,58]. However, the whole matter of food fraud is not a new issue. Already in antiquity, people were scared about commercial fraud or the contamination of food [49,54,58]. One of the earliest and most prominent examples is the Roman law against diluting wine (with water) or refining the taste of wine by adding various sweeteners [59]. Additionally, it has been proven that in the thirteenth century France and Germany passed food control regulation [60].

For preventing food fraud or food crime events, analytical methods are needed to investigate the composition, but also other determinants such as geographical origin of food [38,61].

Before providing an overview of the actual analysis methods, the term “authentic food” has to be defined. A food product is authentic, when the ingredients and the product itself is what it claims to be. More importantly a product is not authentic when the claims, especially the ones that are regulated and written on a product wrapping, are not fulfilled [52,61,62]. Accordingly, authentic food and food fraud are often closely, but not necessarily, connected terms. Authenticity includes for example the species or the geographical origin of food, as well as the production method (e.g., conventional vs. organic, traditional procedures) or processing technologies (irradiation, freezing, or microwave heating), proportion of ingredients, type of raw materials, additives and genetically modified organisms (GMO’s) [52,61,64,65]. The mentioned authenticity categories can be considered as classification methods of food. These can be either binary classification (e.g., processed or not) or n-ary classifications (e.g., n = variable for the numbers of different geographical origins).

ML is a useful tool with regard to a possible classification. ML techniques are applied to perform data processing following the application of analytical methods (chromatography- and MS-techniques) [66,67,68,69,70,71,72,73,74,75]. As mentioned before, mainly PCA [33], PLS-DA [34], SVM [30], RF [35], DT [36], and kNN [37,38] are used. Besides these ML approaches, DL can be used for classification.

A well-known product that is often a matter in food fraud is olive oil. It is either critically discussed in the context of food quality or geographical origin. Many studies deal with this topic and are trying to develop analytic methods to reduce the crime rate in this case. In the following, two papers, which tried to solve that issue with a GC-IMS approaches and the application of chemometric tools are presented. The first study described more commonly used ML-based statistical analytics and the other one shows a novel approach with deep learning algorithms.

In the study presented by Gerhardt et al. (2017), the geographical differentiation of extra virgin olive oil from Spain and Italy was investigated [76]. Researchers used gas chromatography-ion mobility spectrometry (GC-IMS) as an analytical tool to obtain MS data, being treated afterwards with chemometric tools like PCA, LDA, and kNN. To visualize the different geographical origins of the investigated samples a PCA was performed. For the classification of the samples, LDA was applied. As a second method kNN was performed with the aim of classifying. The classification is achieved by finding the nearest samples (k = 5) to an unknown class in the dataset.

Gerhardt et al. showed that after the application of PCA-LDA and kNN classifier, the classification with regard to the origin (Spain vs. Italy) of most of the samples was correct. In that study, it was concluded that GC-IMS coupled with multivariate statistics is a fast screening tool and a great possibility, to deal with the authenticity issue of extra virgin olive oil [76]. The results obtained in that study were only representative for the harvest season 2014–2015, which is a certain limitation of the model. The research gives perspectives to apply DL algorithms, even when the used ML tools showed good results. The application of DL in authentication issues as e.g., in the differentiation of more than two countries of origin offers flexibility to adjust a wider range of samples [76].

In contrast, Vega-Márquez et al. (2020) used DL to classify olive oil with regard to other quality parameters. For classifying olive oil, Vega-Márquez et al. applied DL approaches to process the MS data [77]. They used a set of 701 samples to discriminate three classes (extra virgin olive oil vs. virgin olive oil vs. lampante olive oil). The learning model applied was a FNN. Researchers performed binary classifications (e.g., lampante oil vs. non-lampante oil) as well as a ternary classification of the oils and used different numbers of hidden neurons. The binary models showed better results than the ternary model [77]. A comparison of the ternary classification with an ML application like PCA and OPLS-DA with an accuracy of 74.3%, and the DL approach with an accuracy of 81.4% highlighted that the novel technique was a useful tool in view of the classification of olive oil [77]. The success of the model presented by Vega-Márquez et al. (2020) also revealed some limitations. The available amount of data for training an algorithm is an important aspect for a successful model. In that study, only 701 samples have been available for the development of an algorithm. Therefore, it can be beneficial to use additionally synthetic data [77].

An example for the determination of the geographical origin with the application of DL is described by Long et al. (2017), their focus was on the differentiation of the origin of white rice (*Oryza sativa* L.) samples [78]. In that study, a rapid, accurate, and reproducible method was developed for a differentiation of the geographical origins of white rice (*Oryza sativa* L.) from Korea and China. Researchers identified lysophospholipids as marker compound [78]. Whereas the two papers before used GC-IMS, Long et al. approached the issue with a direct infusion-electrospray ionization-multiple reaction monitoring-mass spectrometry application [79], three different approaches (SVM, RF, and NN) were applied and compared afterwards [78]. DL classification was performed with a deep FNN model. It was established with the use of only 60 white rice samples from the cultivation year 2014. They trained the model with an input layer, four hidden layers, containing 200 neurons per layer, as well as an output layer. The validation took place with a five-fold cross-validation and moreover, with two independent batches of white rice samples which were cultivated in 2015. The results indicated that DL algorithms provided an effective framework regarding the authentication of white rice, or to be more precise, to validate the geographical origin of the product. The research proved that DL is in no way inferior to ML applications [78].

The beforementioned studies showed the application of non-image-based DL algorithms regarding to food authenticity issues [77,78]. Additionally, there was a promising study which applies DL on the processing of metabolomics MS/MS data to identify unknown metabolite-compounds [80]. The metabolic profile of food is a target to evaluate the authenticity of food. The study described by Ji et al. (2019) showed great potential for solving analytic issues although it was not yet applied in a food-related context. Researchers introduced DeepMASS (deep MS/MS aides structural-similarity scoring), which is able to recognize parallels in the structure of unknown metabolites against well-known metabolites in databases depending on their MS/MS spectra [80].

The model converts MS/MS spectra pairs into vectors and takes them with the associated fast Fourier transform cross correlations as inputs and passes them through a layer of neurons. The *m/z*, the mass difference between the pairs and the chemical formulas are also passed as input through a layer of neurons.

The outputs are coupled and provide the input for two additional layers of neurons. With this model, potential metabolite/marker candidates can be predicted, and a mass score is calculated. By integrating a so called fingerprint score (dice correlation of their molecular fingerprint), ranking of potential candidates is revised. When using the model, users are not only able to identify unknown metabolites with a novel and an open-source tool, but also train their own model with the source code provided [80].

## 5. Prediction of Shelf-Life

The prediction of foods’ shelf-life is an important topic in food safety, but also with regard to food security and the aspects of sustainability. In view of a growing world population, it can be challenging to provide safe, affordable, and fresh food for all people on this planet in the future [81,82]. Over the last decade, the strategy to nourish the growing population was to increase the agricultural productivity [81,83]. According to the United Nations, the world population will increase to 9.8 billion people when reaching the year 2050. Considering this prospective, the agricultural productivity needs to be increased by 30–40% for providing an adequate food supply for the whole population [80]. The increase of productivity might not be continuously upscalable in the future, because of the limited environmental and economic resources [84]. The optimal use of the earth’s resources can be a different strategy to maintain an adequate supply for the growing population [84]. The optimal use can be also preventing (food) waste and further food-related losses [84]. Methods to reduce the food waste can be distinguished in two categories. On the one hand, the prolongation of the shelf-life such as improving storage conditions and on the other hand, a more accurate prediction of shelf-life [84,85,86]. Especially, the use of best-before dates for certain food products is one of the main reasons of a significant amount of food wastes in supermarkets [85]. Further, the average consumer most likely throws away food after the best-before date, even when the product is still edible. However, responsibility of setting the best-before date is in the hand of the manufacturers and it is not always clear which parameters are taken into account.

Shelf-life is defined as the time period during the food products preserve a certain level of quality comprising sensory, chemical, physical, and microbiological properties under recommended storage conditions. This means that the product is warranted to be absolutely safe for consumers’ health [82,84,87,88,89,90,91,92]. However, this does not necessarily mean that marketability and quality expire immediately. The manufactures are responsible for maintaining the conditions during the best-before date. Consequently, most of the food products are enjoyable even after the best-before date and fulfill the consumers expectations [93].

Shelf-life of food is primarily determined by sensory and microbiological methods [94,95,96]. Traditional microbiological tests for evaluating the shelf-life demonstrated in the past that the results are of little value to predict the time period in which food “really” expires. This means a risk remains that the food is expired and consequently an ambiguous consumer safety [92,97]. Due to the imprecise determination of the shelf-life, manufactures set the best-before date with a safety margin to prevent any damage to the consumers’ health. The analytic methods and the data treatment afterwards, to determine shelf-life are versatile, but continuously revised. However, chemometric tools like PCA can be used for the prediction of shelf-life and have been already introduced by different research.

Three exemplary studies are presented in the following that predicted shelf-life by combining MS and chemometric tools for data treatment. However, DL is not yet performed for the prediction of shelf-life, but the studies presented can give an impression on what is currently used to treat MS data regarding shelf-life and show potential applications where DL could be used as an alternative. These researchers primarily used ML approaches such as PCA, OPLS-DA and PLS.

Frank et al. (2020) focused on changes in the metabolome (volatile and non-volatile metabolites) in vacuum packaged chilled beef (VPCB) as well as the study of potential shelf-life markers.

In that study, a sensory panel including ten untrained assessors was recruited to describe VPCB based on its appearance, color, and flavor during as well as after the implementation of a storage experiment. Additionally, the total lactic bacteria and total aerobic plate counts were analyzed and evaluated. Non-volatile metabolites were analyzed with a targeted liquid chromatography-triple quadrupole-mass spectrometry approach, while the volatiles were measured with solid phase microextraction gas chromatography-mass spectrometry and proton transfer reaction mass spectrometry. The evaluation of the acquired data was performed with different statistical analysis methods [98,99]. On the basis of the mean volatile and non-volatile contents, a PCA was applied to model differences between the replicate VPCB at different time points. They observed that as the storage time increased, the concentration of most non-volatile metabolites increased as well. In the PCA, the volatile metabolites showed that over the time the concentration increased in total. Although no marker indicating the spoilage of VPCB was identified, differences between VPCB were noted, depending on different storing periods [98].

In the following, a study is presented that evaluated the oxidation products in the metabolome to differentiate the quality of meat during a defined storage period: Rocchetti et al. (2020) studied the metabolic profile of pork patties which were prepared with different antioxidants [100]. On the one hand, they added the traditionally used antioxidant butylated hydroxytoluene (BHT) to a pork patty and compared the metabolome with the results they achieved with pork patties that were prepared with guarana seed extracts (GSE) as a natural alternative. The metabolite analysis was performed with an untargeted approach based on ultrahigh-performance liquid chromatography coupled with electrospray ionization quadrupole time-of-flight mass spectrometry (UHPLC-ESI-QTOF-MS). As a chemometric tool, they used orthogonal projections to latent structures discriminant analysis (OPLS-DA). The OPLS-DA was applied to investigate the main differences between meat samples during 18 days of storage. With the score plot from the OPLS-DA it was possible to achieve a good separation between different meat samples and identify changes of the stored samples. The main differences could be assigned to glycerophospholipids and fatty acids as marker compounds, along with specific compounds deriving from lipid oxidation and protein degradation [100].

While those studies were about meat, Marsili (2000) presented a method for the shelf-life prediction of milk [101] using solid-phase microextraction, MS, and multivariate analysis (SPME-MS-MVA) [101]. For the experiment, pasteurized and homogenized reduced-fat milk (2% milk fat) and chocolate milk (3.5% milk fat) were used and sampled over a time period of 7 month. The focus of the study was volatile bacterial metabolites, which were extracted with SPME and determined with GC-MS. The shelf-life of reduced fat-milk and chocolate milk was predicted separately with partial least-squares regression (PLS). Therefore, the data were split in two parts. One part was used for developing the calibration (64 samples of the reduced-fat milk, 53 samples of the chocolate milk). The other part (20 samples of each) was used for the validation of the model. Using PLS the shelf-life of reduced-fat milk was predicted with an accuracy of ± 0.62 day and for chocolate milk of ± 0.88 day. Additionally, PCA was used for the classification of samples with off-flavors. In that case, off-flavors were artificially caused by spiking the milk with bacteria, cooper-sulfate, and sanitizer. When using PLS, the shelf-life for copper- and sanitizer-contaminated samples could not be predicted accurately, but with PCA researchers were able to distinguish between all different samples [101].

For the prediction of shelf-life, a wide variety of analytical methods are performed, offering the basis for improved chemometric evaluations. The following three examples should illustrate the diversity in this research field. Studies presented were selected with regard to their use of different combinations of analytical strategies and chemometric tools. The earliest published study by Gómez et al. (2008) presented an approach for monitoring the shelf-life of tomatoes (*Lycopersicum esculentum*) with the application of an electronic nose (e-nose) technology, PCA, and linear discriminant analysis (LDA) [102]. Researchers were able to show that PCA and LDA were capable of distinguishing the different tomato storage times of the tomatoes which were stored in a box, as well as the samples which were stored in a bag [102]. Gaggiotti et al. (2019) presented a methodology to evaluate the shelf-life of vegetables (i.e., carrots) based on aromatic compounds with a ZnO-peptide-based quartz crystal microbalances array of gas sensors [103]. There, the carrots were stored at different temperatures for a month. Additionally, to the sensors, GC-MS was used to verify the results and identify volatile compounds. PCA was conducted to verify the sensors’ ability to discriminate between the storage temperatures and by that the shelf-life. Researchers used PCA as an unsupervised multivariate data analysis. They gained promising results by using it in combined sensors [103].

A research investigating the shelf-life life of extra virgin Argan oils (*Argania spinosa* L., EVAO, being produced with Argan fruits harvested in Morocco in 2014 and 2015) is described by Kharbach et al. (2021) [104]. Two different procedures have been applied in the production of the EVAO. Half of the seeds were roasted, the other half remained unroasted. The other procedure was differentiating the light transmission of the used bottles for storage (clear vs. dark bottles). Over a time period of two years all samples were analyzed each 6 months of storage. For the analysis, the main chemical properties (acidity, peroxide value, fatty acids etc.,) were determined and Fourier transform infrared spectroscopy (FTIR) was used. In this case, chemometrics was applied to investigate the influence of the storage period and the packaging material, influencing the shelf-life of EVAO. PCA and PLS-DA were performed to distinguish between fresh and oxidized oils based on the chemical properties and the FTIR data. Those approaches allowed evaluating the EVAO oxidative quality during storage. Authors concluded that FTIR spectra and chemometric tools were a well combination to study the shelf-life of EVAO [104].

For reducing wastes of food successfully and sustainably prediction of the best-before date needs to be far more accurate. A possibility to fulfil this need can be a combination of MS and DL algorithms. As described above, there are already studies that applied DL at hand of MS-based data [77,78]. However, a bunch of MS-based approaches in a medicinal context exist as well [105,106]. These applications can be adapted to the issue of (food) shelf-life prediction in future studies. In the context of the determination of food shelf-life, efforts to treat data with AI-based soft-sensors have been made [107]. However, the combination with an omics-based technology or to be more precise a MS-based data is not yet performed.

In a study described by Fathizadeh et al. (2021), the classification of shelf-life of apples was predicted. They performed the study with vibration sensors using fast Fourier transformation, a following pattern recognition technique, and an artificial NN [108]. Furthermore, efforts have also been made to investigate the proteome during storage to identify peptide markers of different foods, e.g., milk products and oysters. These identified peptide markers are possibly able to reflect the shelf-life [109,110,111].

However, proteomic approaches for shelf-life prediction also offer a basis for the integration of DL. A technique that can be applied is the combination of peptide sequencing and DL. There are interesting approaches in de-novo sequencing using this type of learning. It can potentially be used for the identification of biomarkers, for determining the shelf life of foods, but also many other food-related research questions.

## 6. Peptide Sequencing

The proteomic approaches in food authentication and shelf-life evaluation were so far less addressed than the metabolomic approaches, although proteins are one of the main compounds in foods. To identify proteins in food, the amino acid sequence has to be determined, even if it is only on a peptide level. These peptides can be also used as biomarkers to solve different tasks in foodomics like differentiating between species [112,113] or the kind of rearing system in animal production [114].

There are two approaches for using peptide sequencing with tandem-mass-spectrometry data: database search methods and de-novo sequencing. The first approach uses database search engines such as Mascot [115], PEAKS DB [116], SEQUEST [117], and MaxQuant [118]. Therefore, the sequencing accuracy depends on the extent of the database used. The de-novo technique is simply based on the generated spectra, making it possible to sequence not database-registered proteoformes. The sequencing is based on the observed mass- signals and the mass differences in between. Those differences are caused by the fragmentation of the backbone of the peptide, resulting in fragments like b- and y-ions [119]. Based on the *m/z* profile, the sequence is aligned. Challenges like isobaric amino acids have to be considered, as well. Leucine and isoleucine cannot be distinguished based on their mass. A fragmentation of the side chain enables the differentiation of these two due to the resulting fragments [120,121,122]. Moreover, glutamine and lysine are not easy to distinguish either, because of their low mass delta of 0.036 Da. The same is valid for asparagine and the peptide consisting of two glycines, whose mass delta is 0.000002 Da [123]. Due to the low mass difference of some peptides the fragment coverage of the spectra is key for the accuracy of the sequencing process. Novor [124], PepNovo [125], or PEAKS [126] are examples for available de-novo-sequencing software.

With regard to data evaluation, peptide sequencing can be seen as a classification, with the 20 main canonical amino acids as the classes. Every amino acid has to be classified one after the other. When taking amino acid modifications into account as well, the classification of a peptide is more complex and reveals why proteoforme analysis is an emerging, but still very challenging approach, finally providing lots of data [9].

In 2017, Tran et al. applied DL in the context of de-novo peptide sequencing data by introducing DeepNovo [127]. They used two types of NN—CNN and LSTM—to predict each amino acid at a time. When taking a look at the structure of their algorithm, in the first step the MS spectrum is transformed into an intensity vector which contains the *m/z* as indices and the intensity as values. A so-called spectrum CNN and a LSTM learn sequence pattern of amino acids based on the spectrum.

The sequencing itself is divided into two parts: in the first part, the prefix mass is calculated. The prefix mass is the sum of the masses of the amino acids that are predicted up to that point. Then the mass of each amino acid with its calculated b- and y-ions is added. It considers the 20 proteinogenic amino acids as well as some prominent modifications like deamination. For each fragment, a window is calculated that includes masses 0.5 Da smaller and bigger than the expected mass. All these information are processed in an ion-CNN. Second, a LSTM is integrated that processes a given prefix as an embedding vector. The outputs of the CNN and the LSTM are combined. Dynamic programming and probability distribution follow to make the final prediction [127].

In 2019, Tran et al. expanded their model for data-independent acquisition (DIA) data [128]. DIA-data is more complex, as one precursor can be associated with more than one spectrum as well as a spectrum can contain fragments from different precursors. DeepNovo-DIA rearranges the spectra according to their precursor. The precursor and the associated spectra are fed into the network. The network contains ion-CNN, spectrum-CNN, and LSTM to achieve three main aspects: learning the 3D shape of the fragments, the relation between the precursors, and the fragment ions and the peptide sequence pattern [128].

pNovo3 is a different approach for the integration of DL for peptide sequencing [129]. It is based on pNovo+, a de-novo sequencing algorithm which delivers the top ten peptide candidates [130]. Afterwards these candidates are reranked, starting by generating theoretical spectra with pDeep [131]. pDeep is a DL algorithm using biLSTM (bidirectional long short-term memory) for the prediction of MS/MS spectra of peptides. From database statistics and the theoretical spectra features are extracted. The similarities are calculated, and a learning-to-rank-framework and spectrum merging are applied. Based on their outputs the top-1 result is predicted [129].

De-novo sequencing can be applied as an alternative to database-driven approaches. It enables the characterization of peptides and proteins of food components beyond the known and already sequenced proteins.

Peptides can be used as biomarkers for food authentication as it can be seen by the three following research [132]. For example, peptide biomarkers can be applied for meat species identification according to Sentandreu et al. (2010) [133]. They identified species-specific peptide markers for chicken for detection in various meat mixtures. An example of peptide biomarkers in meat adulteration is the study described by Leitner et al. (2006) [134]. In that study, soybean peptide markers in meat products were identified. Soybean protein was added to meat in order to enhance functional properties [134]. A third example is from the field of food allergenicity: Chaissange et al. (2007) used peptide markers to identify residual allergens in processed peanuts [135]. These three examples all used databases to identify the peptide markers.

Carrera et al. (2007) performed de-novo sequencing of fish species of the family *Merlucciidae* for characterizing the species [136]. They performed de-novo sequencing, as the amino acid sequence of the proteins was not available in databases. That study demonstrates that the tool of de-novo sequencing can be needed for the identification of peptide biomarkers, because not all proteins are yet available in databases [136]. In general, de-novo sequencing is a powerful tool in proteomics to address major challenges in foodomics like the identification of food (protein) components, the study of changes due to processing, as well the study of issues of food authentication and food safety [65,137]. In addition, the integration of DL provides the potential of further improving this promising tool.

## 7. Conclusions and Future Perspectives

Although DL is an emerging technique, application of DL in mass-spectrometry is still a minor research field in foodomics, which is underlined by the limited number of research. Nevertheless, it is a field with large potential of all kinds of questions related to food, food quality, food safety, and food security. In comparison with ML, DL does not require manual feature extraction. DL automates the tuning of parameters. It is superior when using with large datasets, especially using unstructured data. Deep neural networks are applicable to versatile assignments [31,39,41].

Food authenticity is a central research question in foodomics, comprising also the challenges mentioned above. First steps in the direction of establishing DL applications have been made and as mentioned, research done so far showed promising perspectives. The number of applications has to be extended to a broader field of matrices and research questions.

Shelf-life is a field that has potential for the application of DL, especially because of the easy handling of high throughput data, when using mass spectrometry-based analysis models. Although, DL has not yet been used with regard to MS-data in this topic. Recent research in the field of food authentication showed that the novel DL approaches can compete with traditional mathematical methods. The traditional methods take longer and are not able to handle the large amount of data easily [78].

The integration of DL in de-novo peptide sequencing provides perspectives for foodomics, as well. DL is already used in studies for de-novo peptide sequencing. It has not been integrated into foodomics, so far. However, the countless number of food-relevant proteins/peptides might bear the problem that database approaches can be insufficient and de-novo peptide sequencing needs to be done anyhow [65]. Proteomics already uses DL algorithms for the prediction of retention time [138] or MS/MS spectra [131,139,140]. These can be implemented into the field of food analysis to accomplish different tasks in foodomics.

A limiting factor for DL applications is the number of available training data. Collecting the necessary data is challenging for single research groups. Wen et al. [141] proposed a collaboration between data scientists and experimentalists could make training data far more accessible in appropriate scales [141].

A field of DL that has potential of success in solving issues in foodomics is *Deep Reinforcement Learning*. It can generalize better, especially in unknown environments. Therefore, it has potential for the area of food authenticity.

The datasets for these applications are normally limited by factors like years or geographical origins and these limitations are imported into the DL algorithm when using supervised learning [39,41,142].

Moreover, the application of DL as well as ML needs some knowledge in programming. Due to the importance of chemometrics in food analysis, informatics needs to be implemented into education and training of food chemists/food analytics.
